# Detection of Geothermal Anomalies in Hydrothermal Systems Using ASTER Data: The Caldeiras da Ribeira Grande Case Study (Azores, Portugal)

**DOI:** 10.3390/s23042258

**Published:** 2023-02-17

**Authors:** Jéssica Uchôa, Fátima Viveiros, Rafaela Tiengo, Artur Gil

**Affiliations:** 1 Research Institute for Volcanology and Risks Assessment (IVAR), University of the Azores, 9500-321 Ponta Delgada, Portugal; 2 cE3c—Centre for Ecology, Evolution and Environmental Changes, Azorean Biodiversity Group, CHANGE—Global Change and Sustainability Institute, Faculty of Sciences and Technology, University of the Azores, 9500-321 Ponta Delgada, Portugal; 3 Faculty of Sciences and Technology, University of the Azores, 9500-321 Ponta Delgada, Portugal; 4 Departamento de Ingeniería Agroforestal, ETSIAAB—Escuela Técnica Superior de Ingeniería Agronómica, Alimentaria y de Biosistemas, Universidad Politécnica de Madrid, 28040 Madrid, Spain

**Keywords:** thermal anomalies, remote sensing, hydrothermal system, geothermal

## Abstract

Current-day volcanic activity in the Azores archipelago is characterized by seismic events and secondary manifestations of volcanism. Remote sensing techniques have been widely employed to monitor deformation in volcanic systems, map lava flows, or detect high-temperature gas emissions. However, using satellite imagery, it is still challenging to identify low-magnitude thermal changes in a volcanic system. In 2010, after drilling a well for geothermal exploration on the northern flank of Fogo Volcano on São Miguel Island, a new degassing and thermal area emerged with maximum temperatures of 100 °C. In the present paper, using the ASTER sensor, we observed changes in the near-infrared signals (15 m spatial resolution) six months after the anomaly emerged. In contrast, the thermal signal (90 m spatial resolution) only changed its threshold value one and a half years after the anomaly was recognized. The results show that wavelength and spatial resolution can influence the response time in detecting changes in a system. This paper reiterates the importance of using thermal imaging and high spatial resolution images to monitor and map thermal anomalies in hydrothermal systems such as those found in the Azores.

## 1. Introduction

Sudden thermal changes in a volcanic system may constitute a precursor sign of eruption [1]. A recent study [2] highlighted that subtle long-term increases in heat flux could precede magmatic and phreatic eruptions. These observations demonstrated the importance of continuous and broad monitoring of active volcanic areas. Remote Sensing (RS) has been playing an important role in observing these systems [3], such as monitoring programs AVO [4], MODVOLC [5], HOTSAT [6], HOTVOLC [7], AVHotRR [8], VoltSatView [9], RSTVOLC [10], MIROVA [11], HOTMAP [12], AVTOD [13], MOUNTS [14], and NHI [15].

Several studies have applied RS techniques to study high temperature (>100 °C) fumarolic fields (e.g., [16,17,18]); however, the identification of hydrothermal (<100 °C) fumaroles has been challenging [19]. Nevertheless, a few studies have already shown the use of satellite data to recognize anomalous thermal zones in hydrothermal systems, such as the studies carried out at the Nisyros (Greece) [20] and Solfatara (Italy) [21] volcanoes. Changes in the hydrothermal system of the Yellowstone Volcano (USA) were detected through thermal images in 2007; however, visible spectrum images allowed for the first signs of change in vegetation to be identified in 2001 [22] using high-resolution images. This case highlights the relevance of associating thermal and visible data for monitoring remote areas.

The fumarolic fields found in the Azores archipelago show maximum temperatures around 100 °C, similar to the fumaroles that characterize the above-mentioned volcanic areas. At Fogo Volcano, on São Miguel Island, manifestations of secondary volcanism comprise hydrothermal fumaroles, thermal and cold CO_2_-rich springs, and diffuse degassing areas. In addition, a geothermal exploration area is located on the volcano’s northern flank [23].

In this research, Advanced Spaceborne Thermal Emission and Reflection Radiometer (ASTER) sensor data were used to understand the sensor’s sensitivity in detecting changes that occurred after the emergence of a thermal anomaly next to a geothermal well in the Fogo Volcano area.

This anomaly appeared in 2010 after drilling a geothermal well near the Caldeiras da Ribeira Grande site on the northern flank of Fogo Volcano, accompanied by the progressive burning of vegetation. Considering that this was the first significant thermal change in the Azores archipelago after the launch of the ASTER, this study aims to evaluate the response time of this sensor in the near-infrared, red, and thermal infrared, considering the different spatial resolutions.

Due to the difficulty in quantitatively measuring the surface temperature due to pixel saturation problems or atmospheric attenuation, some studies are based on determining pixel value thresholds by isolating the background temperature found in each image. The anomalous pixel value may be above average plus a standard deviation (σ), as in [24], above average +2σ [25], or with multiple thresholds [26], since a maximum temperature in one image can represent a normality scenario in another [16,27].

In this work, we evaluated the ASTER data from 2010 to 2012 to monitor the growth of the fumarolic field and the sensor’s response time.

### Research Constraints and Challenges

Some thermal sensors have been used for monitoring temperature in volcanic systems, such as the Meteosat Second Generation-Spinning Enhanced Visible and InfraRed Imager (MSG-SEVIRI); the Advanced Very High-Resolution Radiometer (AVHRR); the Moderate Resolution Imaging Spectroradiometer (MODIS); the Advanced Spaceborne Thermal Emission and Reflection Radiometer (ASTER); the Landsat; the Sentinel-2; and the Sentinel-3 (e.g., [15,28]). The ASTER is a sensor with thermal bands that has the highest spatial resolution among open-source sensors. However, its average temporal resolution is 16 days, but its ability to be directed to areas of interest may result in an increase or decrease in this interval [29].

Applying RS techniques to detect thermal anomalies is particularly challenging in low-temperature areas. Several factors may interfere with detecting thermal anomalies using remote sensing, such as the effect of sunlight, which can mask thermal anomalies. Consequently, nighttime images should minimize this effect by isolating thermal anomalies from the background temperature [26]. Urban areas can also mask thermal anomalies since they are responsible for heat island effects. Land-use maps are thus relevant tools that complement the produced thermal maps [25].

Another critical factor is the relationship between the temperature of a thermal anomaly and the brightness temperature measured in a pixel, which is directly related to the fraction of a pixel occupied by this anomaly [19,30]. Low-temperature thermal anomalies, such as the hydrothermal systems found in the Azores, constitute an important challenge for remote thermal sensing.

## 2. Study Area

São Miguel Island is one of the nine Azorean volcanic islands (Portugal). The island comprises seven volcanological units, including the Fogo Volcano [31]. The Fogo Volcano is a polygenetic system, with secondary volcanism manifested in cold CO_2_-rich and thermal springs [32,33,34], low temperature (<100 °C) fumarolic fields, and diffuse degassing areas, essentially located on the northern flank [35].

**Figure 1:**
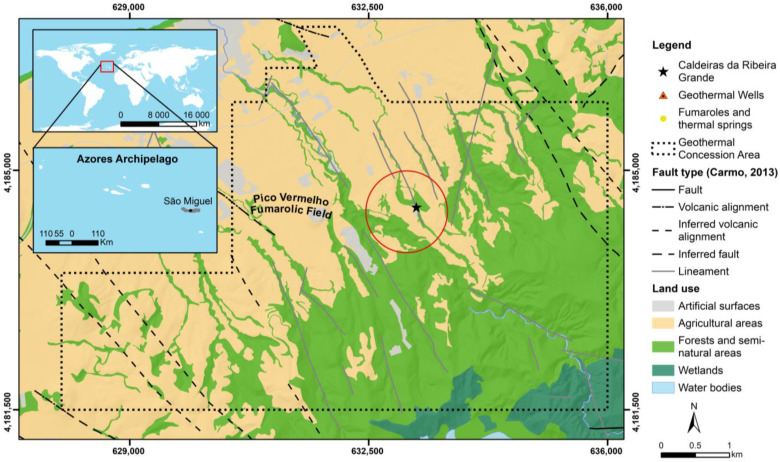
Land-use map (based on [37] and the geographic location of the Ribeira Grande geothermal field) with the existing geothermal wells (based on the studies of [23,38,39]), the main fumarolic emissions [35], and fault type (based on [40]).

In 2010, after drilling a well (RG4) for geothermal energy exploration close to the Caldeiras da Ribeira Grande site (Figure 1), a new degassing area emerged that was accompanied by a thermal anomaly with a maximum temperature of 100 °C [36].

**Figure 2:**
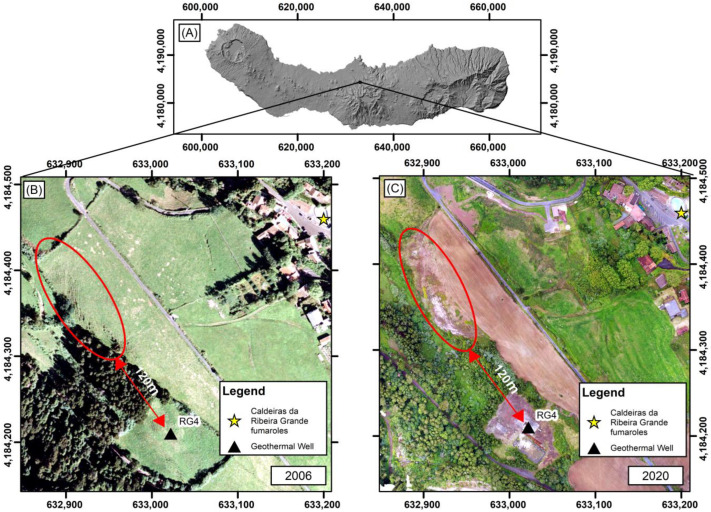
Location of the study area highlighting the surrounding area of the RG4 well (signed as a black triangle). (**A**) São Miguel DEM; (**B**) orthophoto map of the RG4 surrounding area in 2006, before the thermal anomaly; and (**C**) UAV RGB image after the thermal anomaly, 2020 (Source: CIVISA). The red line corresponds to the area with the highest concentration of anomalies and visible alteration of vegetation.

According to the authors of [36], in 2009, the drilling of the RG4 geothermal well began in an area near the Caldeiras da Ribeira Grande, reaching about 470 m in depth. However, the drilling intercepted a superficial aquifer at about 230 to 250 m, which conditioned its execution. It was posteriorly sealed and abandoned in February 2010. Before February 2010, the only existing gas emission and thermal anomaly in the area were circumscribed to Caldeiras da Ribeira Grande fumarole (highlighted as a star in Figure 1). Gas and temperature measurements began periodically in the area after February 2010, and a new fumarolic field (with maximum temperatures around 100 °C and hydrothermal compositions) developed in the area after May 2010 (Figure 2). Since early 2012, the spatial distribution of the main gas emissions (essentially CO_2_) and anomalous thermal areas remained quite stable [41].

This study focused on the anomaly discussed above since it offered a new opportunity to explore an anomaly using RS-based approaches. As mentioned above, it constitutes the first significant anomaly to emerge after the launch of the ASTER, enabling the evaluation of the effectiveness of this sensor for monitoring changes in hydrothermal areas, such as those existing in the Azores archipelago.

**Figure 3:**
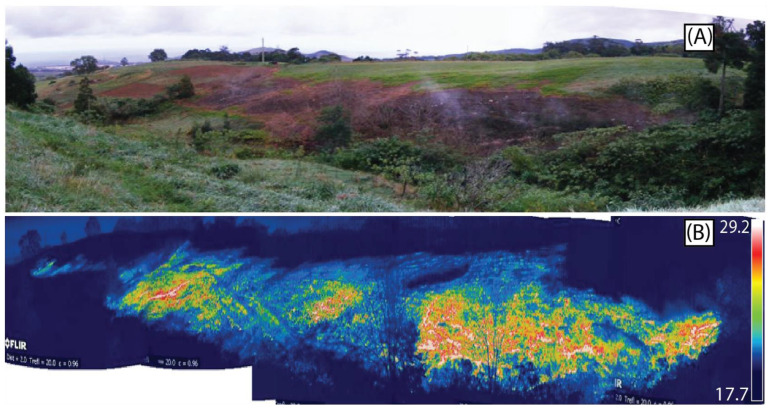
Oblique aerial photo of the degassing area located next to the RG4 well with altered vegetation. (**A**) The visible image shows the altered vegetation associated with the thermal anomaly, and (**B**) the thermal image acquired with a FLIR Therma CAM™ SC640 thermal infrared camera (thermal and visible image provided by IVAR/CIVISA acquired on July 21, 2010).

Figure 3 shows the difference between the background temperature and the anomaly in an oblique thermal image acquired using a FLIR Systems Therma CAM™ SC640 thermal infrared camera in 2010.

## 3. Materials and Methods

Degassing areas with medium or high temperatures are commonly analyzed using RS due to the significant difference between the background temperature and the temperature of fumaroles. However, hydrothermal systems are still a challenge, considering the factors that can interfere with their identification, such as the small difference between the bottom temperature and the temperature of fumaroles.

The ASTER instrument is a high-resolution multispectral space sensor aboard NASA’s Earth satellite launched in December 1999, and it began data acquisition in March 2000 [42]. Table 1 presents the main features of the ASTER instrument [29]. For this work, only spectral bands 2, 3N (for NDVI), and 13 were used. Band 13 was used due to less interference from the atmosphere [43].

The images used in this work (Table 2) were provided by the USGS EarthExplorer platform (https://earthexplorer.usgs.gov/, accessed on 22 February 2022). For the thermal analyses, only nighttime data were used to minimize the effects of topography and surface insolation [25,26,27,44].

It was not possible to use completely cloud-free images in this work due to the significant and almost permanent cloud coverage, which constitutes a relevant challenge for the RS applicability in most small oceanic islands [45], usually resulting in a low number of available images.

The criterion for defining thresholds should be made according to the sensor and available imagery by separating the background temperature. The present work used the brightness temperature, which consists of the radiance obtained by the satellite, representing the temperature of a blackbody emitting radiation [46]. Thus, converting the Digital Numbers (DN) of the image to radiance with Equation (1) [29]:

(1) $$L_{\lambda }=\left(\mathrm{DN}-1\right)\times \mathrm{UCC}$$

where L_λ_ is the spectral radiance; DN is the thermal infrared band digital numbers; and UCC is the published Unit Conversion Coefficient (0.005225 w/m^−2^/sr^−1^/µm^−1^).

Temperature (measured in degrees Celsius) is then given by Equation (2):

(2) $$T_{k}=K_{2}/\ln\left(\frac{K_{1}}{L_{\lambda }}+1\right)-275.15$$

where K_1_ (641.32) and K_2_ (1271.22) are constants derived from Planck’s radiance function.

As demonstrated by the authors of [26], different thresholds may be used in the same image to identify thermal anomalies. This study categorized the thresholds into three classes: below the +2σ mean, above the +2σ mean, and above the +3σ mean for greater clarity of the different temperatures identified in the ASTER data.

It is commonly accepted that degassing can influence vegetation [47]. Thus, the Normalized Difference Vegetation Index (NDVI) was also applied, as it is related to the amount of biomass, vigor, and photosynthetic activity [48,49,50]. NDVI was used to analyze the time required for detecting the anomaly using near-infrared and red (with 15 m spatial resolution). 

The NDVI was calculated using band 2 (red) and band 3 (NIR), as shown in the following expression (Equation (3)):

(3) $$\mathrm{NDVI}=\frac{\mathrm{NIR}-\mathrm{RED}}{\mathrm{NIR}+\mathrm{RED}}$$

The NDVI is an index ranging from −1 to +1. Values close to +1 are commonly related to areas with great vegetative vigor, while −1 is related to ice, and 0 is associated with bare soil. This index has a high sensitivity to changes in vegetation cover, which may be a relevant driver for identifying changes in hydrothermal systems related to vegetation burning, as in the case study. The NDVI ranges were classified into five classes [51,52], as presented in Table 3.

## 4. Results and Discussion

The statistical analysis performed in this work aimed to minimize the effects of data seasonality since the available RS data were not obtained in the same seasons, and the temperature variability was significant throughout the different periods of the year.

**Figure 4:**
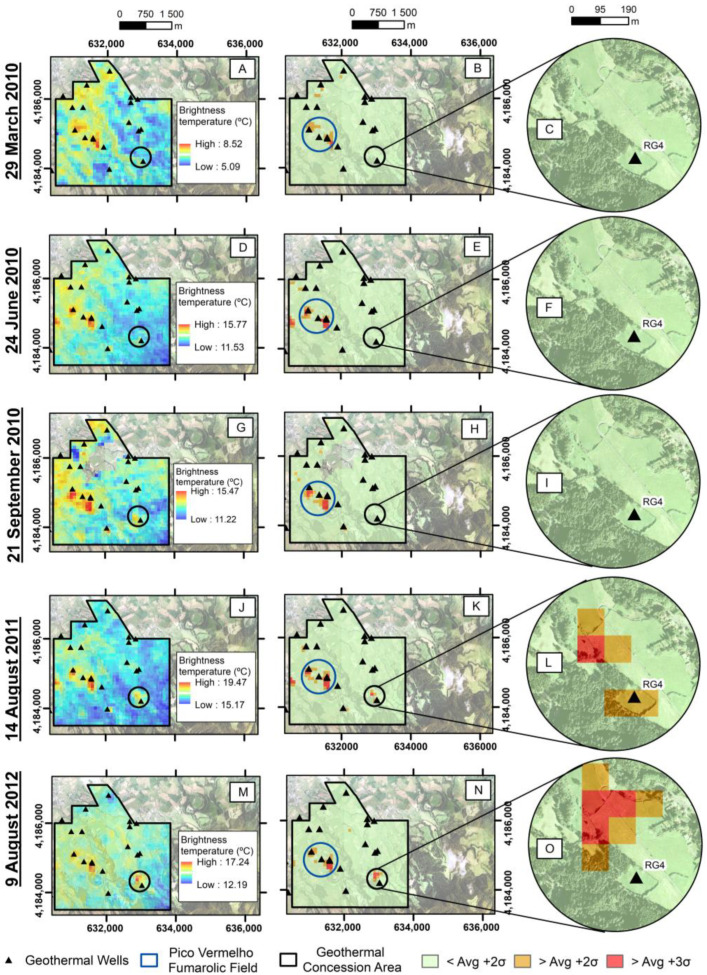
Part of the Geothermal Concession Area: (**A**) the ASTER nighttime thermal infrared image from 29 March 2010, (**B**) normal temperatures (below average temperature + 2σ) presented in green, above average temperatures + 2σ in orange, and above average + 3σ in red, and (**C**) framing of the area near the RG4 well, where it was not possible to identify thermal anomalies; (**D**) the ASTER nighttime thermal infrared image from 24 June 2010, (**E**) normal temperatures presented in green, above average temperatures + 2σ in orange, and above average + 3σ in red, and (**F**) framing of the area near the RG4 well, where it was not possible to identify thermal anomalies; (**G**) the ASTER nighttime thermal infrared image from 21 September 2010, (**H**) normal temperatures presented in green, above average temperatures + 2σ in orange, and above average + 3σ in red, and (**I**) framing of the area near the RG4 well, where it was not possible to identify thermal anomalies; (**J**) the ASTER nighttime thermal infrared image from 14 August 2011, (**K**) normal temperatures presented in green, above average temperatures + 2σ in orange, and above average + 3σ in red, and (**L**) framing of the area near the RG4 well, where it was possible to identify thermal anomalies; (**M**) the ASTER nighttime thermal infrared image from 09 August 2012, (**N**) normal temperatures presented in green, above average temperatures + 2σ in orange, and above average + 3σ in red, and (**O**) framing of the area near the RG4 well, where it was possible to identify thermal anomalies.

Five images covering part of the geothermal exploration area were analyzed. The selected study site (Figure 4) was chosen from the total geothermal exploration area (Figure 1) and aimed at minimizing the influence of the different land uses and altimetry.

The results show that on 29 March 2010, only a few areas near the already ongoing geothermal wells had a brightness temperature above the average +2σ and the average +3σ. It is worth mentioning that on this date, the only problems had been found in the drilling of the well, without significant changes in the soil temperature. Anomalous soil temperatures were detected with in situ measurements in May 2010. Despite the anomalies associated with RG4, other anomalous areas were found, essentially associated with the location of the geothermal wells, confirming the adequacy of this methodology to detect anomalous areas with temperatures up to 100 °C, as measured by the authors of [53,54] in the areas of the wells and the Pico Vermelho fumarolic field. This pattern on the thermal images remained until 14 August 2011, when the ASTER detected an anomaly near the RG4 well area for the first time, showing brightness temperatures above the average +2σ and the average +3σ.

**Figure 5:**
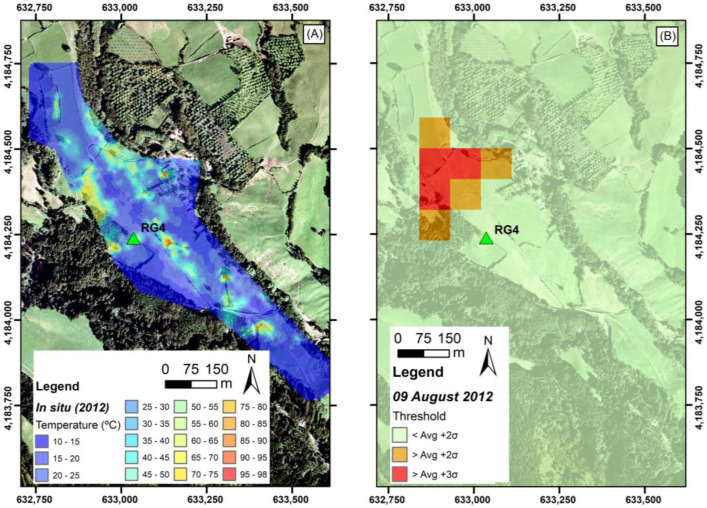
Comparison map with (**A**) in situ soil temperature map (data from January 2012) (Source: F. Viveiros, IVAR/CIVISA, 2012) and (**B**) thermal anomalies on 08 August 2012.

The brightness temperature near the RG4 well remained above the +2σ average and the +3σ average in 2012 and widened in area. Figure 5 consists of the temperatures measured in the soil on January 2012 at about 12 cm depth by F. Viveiros (personnel communication). Figure 5B shows the thermal anomalies found in the image of 08 September 2012.

Despite the seven months between the measurements performed in the field and the ASTER imaging, it is important to highlight that the degassing and thermal anomalies that developed in the field after May 2010 have been quite stable since early 2012 [41]. Nevertheless, as observed in Figure 5A, some of the anomalous thermal zones are smaller than the anomaly that developed in the northwestern area of RG4. This area was mostly detected with the ASTER data (Figure 5B). As mentioned, there are areas mapped in the thermal anomaly map resulting from the field measurements that do not appear in the ASTER image. Some differences could eventually explain this as being due to the interval between the date of the field measurement and the satellite image. However, measurements carried out in 2016 [41] still show the same general spatial distribution of anomalous thermal areas. The most probable explanation relates to the influence of the sub-pixel area [44] since the areas identified by the ASTER correspond to the more extensive in situ degassing area, as shown in Figure 5.

**Figure 6:**
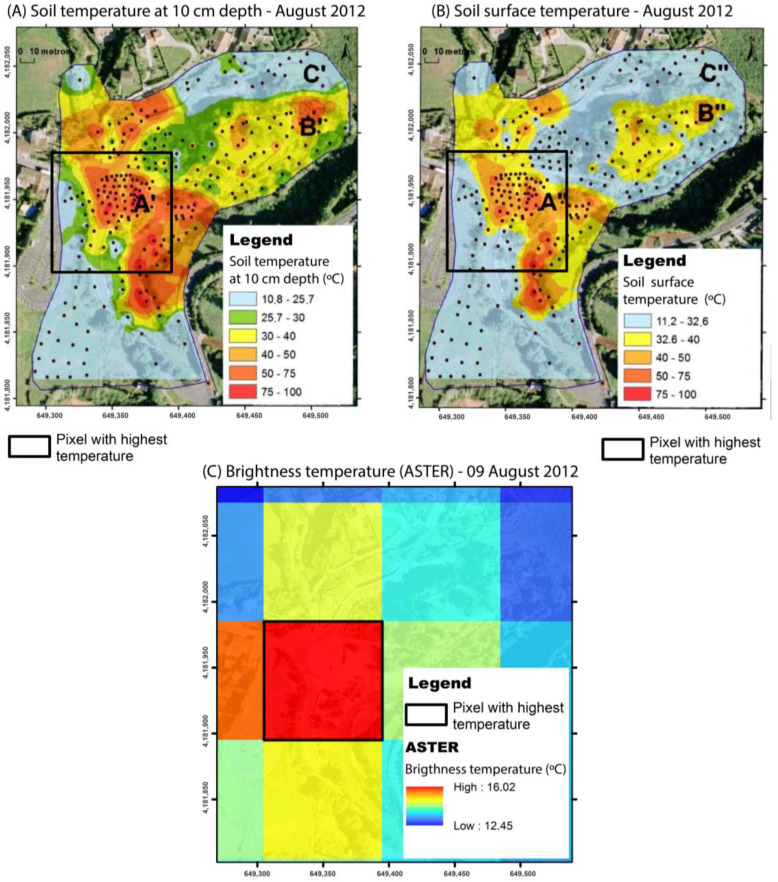
Comparison between temperature (**A**) in the soil at a depth of 10 cm (Pacheco, 2013), (**B**) on the ground surface in situ (Pacheco, 2013), and (**C**) measured using the ASTER sensor in the area comprising the Furnas hydrothermal fumaroles.

Recognition of thermal anomalies is thus highly dependent on the sub-pixel area, and Figure 6 shows one example that may contribute to a better understanding of one of the difficulties involved in identifying thermal zones using satellite data, especially in low-temperature fumarolic fields, as the ones observed in the Azores archipelago.

Figure 6 shows the comparison between the soil temperature at 10 cm depth, the surface soil temperature measured in situ, and the ASTER brightness temperature in a fumarolic field at the Furnas volcano, on São Miguel Island, in August of 2012. It is possible to observe that the pixel with the highest brightness temperature coincides with the area with the highest concentration of thermal anomalies measured on the surface, which shows that a pixel needs to have a large part of its area occupied by an anomaly to allow the detection of the anomaly. This constraint explains the identification of only the major anomalous zone at the Caldeiras da Ribeira Grande site.

**Figure 7:**
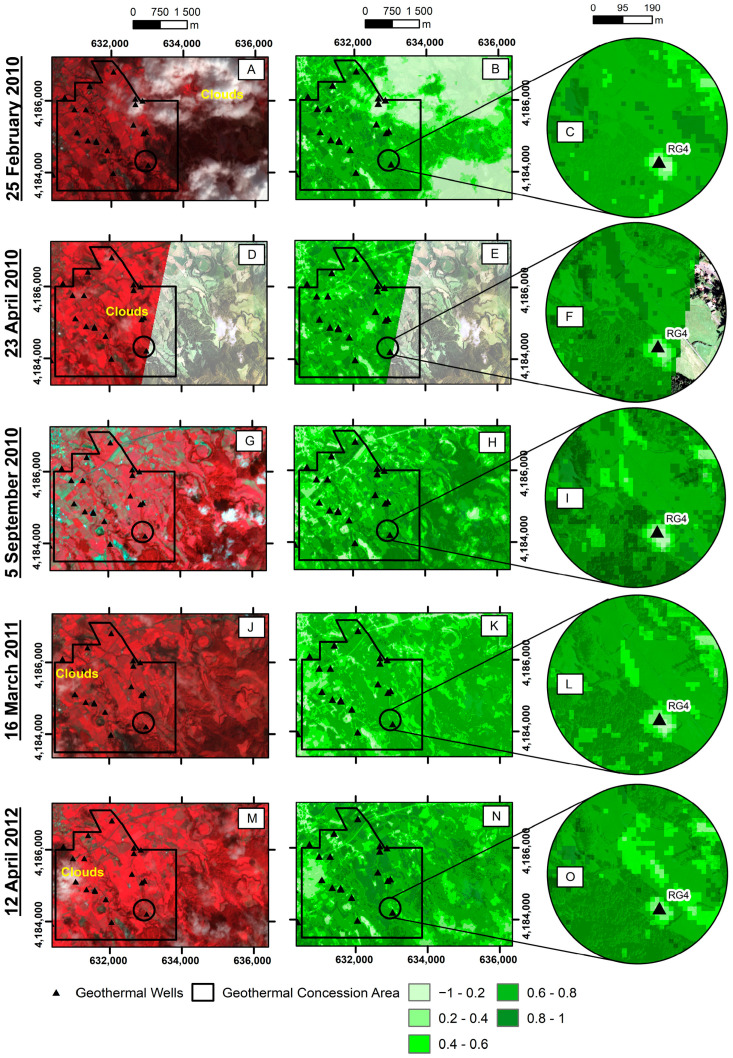
Part of the northern flank of Fogo Volcano: the ASTER RGB composite image (bands 3, 2, 1), the NDVI map of the study area, and framing of the NDVI map near the RG4 well, respectively, for the following dates: 25 February 2010 (**A**,**B**,**C**); 23 April 2010 (**D**,**E**,**F**); 5 September 2010 (**G**,**H**,**I**); 16 March 2011 (**J**,**K**,**L**); 12 April 2012 (**M**,**N**,**O**).

Considering that the thermal anomaly originated changes in vegetation vigor, as shown in Figure 3, an NDVI-based analysis was performed to confirm this evolution (Figure 7). Figure 7 highlights that in February 2010 and April 2010, it was not yet possible to identify changes in vegetation vigor based on the NDVI. This is, in fact, in agreement with the in situ observations since, at that time, no increase in soil temperature had yet been identified; only a drilling problem was diagnosed.

In September 2010, at the end of the summer season (a period with lower rainfall), there was a significant increase in areas with low NDVI values around the RG4 well. Almost a year after, in March 2011, the surrounding vegetation began regenerating with the potential effect of rainfall and the adaption of the plants. However, the anomaly area continued to have low NDVI values and remained so until April 2012.

Precipitation is an important influence factor on thermal radiation [55] and vegetative vigor. The summer on São Miguel Island coincides with lower rainfall values [56]. Nevertheless, the high rainfall index on this island returns an increased vegetative vigor, allowing healthy vegetation to regenerate quickly. However, the area with a thermal anomaly shows an NDVI value below 0.6, even after periods of intense rainfall.

Thus, as in the case of thermal bands, where nighttime images allowed a better separation between hot pixels and background temperature, changes in vegetation vigor associated with thermal anomalies were also easier to identify on NDVI maps created with non-summer images.

**Figure 8:**
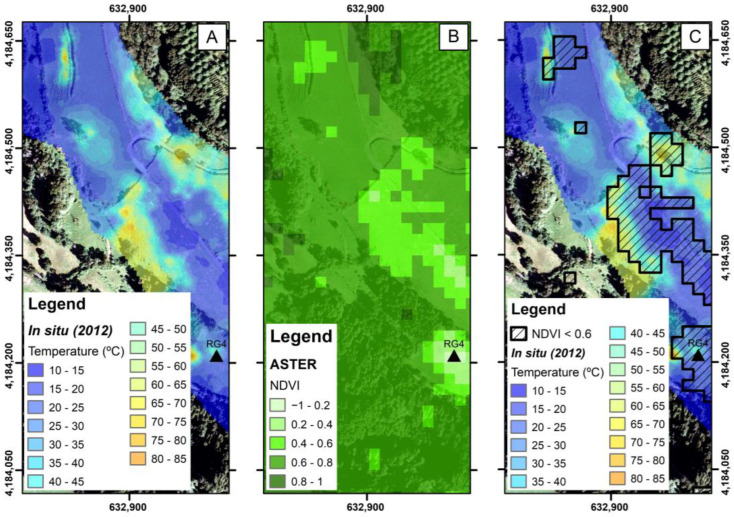
Overlay of the lowest NDVI map values (April 2012) with the respective in situ soil temperatures map (January 2012). (**A**) In situ soil temperature; (**B**) the ASTER NDVI values; and (**C**) a comparison of the soil temperature and lowest values of the NDVI.

According to the Köppen Climate Classification, the temperate and rainy climate of the Azores enhances vegetative vigor. This factor also highlights the relevance of the current study since, despite some effect of seasonality on the vigor of natural vegetation, the maintenance of low NDVI values can constitute important signs of permanent change on the surface related to the deep-derived contribution, as evidenced in volcanoes such as Yellowstone [22] and Hawai’i [57], where alterations in vegetation vigor due to thermal anomalies were detected. Identifying and understanding the land use dynamics in the study area are essential to minimize evaluation errors as mentioned above. For instance, in our study area that was dominated by pastureland, changes in vegetative vigor might also have been related to drought [58], use of herbicides [59], and grazing [60,61]. Therefore, we overlaid the pixels showing the lowest NDVI values of 12 April 2012, with the respective in situ temperatures (measured on January 2012 at 10 cm depth), as shown in Figure 8

Figure 8 shows that areas with thermal anomalies were generally associated with NDVI values below 0.6. Although the ideal would be to compare both datasets acquired on the same date, even with a three-month difference, it is possible to observe a noticeable overlap between areas with higher soil temperatures and lower vegetative vigor.

**Figure 9:**
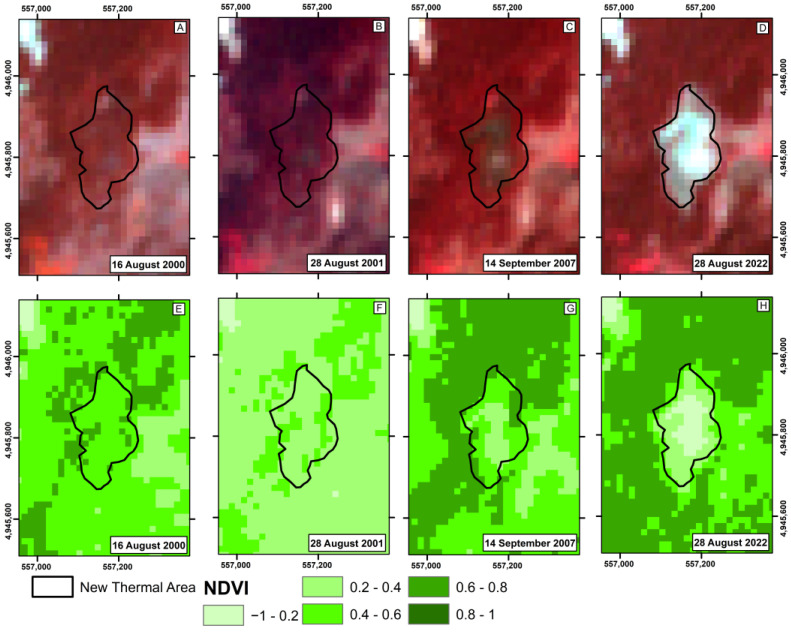
Yellowstone new thermal anomaly near Tern Lake: the ASTER RGB composite image (bands 3, 2, 1) and the NDVI map, respectively, for the following dates: 16 August 2000 (**A**,**E**); 28 August 2001 (**B**,**F**); 14 September 2007 (**C**,**G**); 28 August 2022 (**D**,**H**). The black line corresponds to the new thermal anomaly area.

To verify the pertinence of using the NDVI for another hydrothermal system, the thermal anomaly documented by the authors of [26] at the Yellowstone volcano was checked. Figure 9 shows the vegetative vigor measured from the NDVI with the ASTER before and after the appearance of the aforementioned anomaly.

The thermal anomaly in the Yellowstone volcano hydrothermal system was first identified in 2019. However, when investigating historical images, the authors of [22] found that in nighttime thermal images, the anomaly only significantly changed in radiation in 2007. In visible images, it was observed that the vegetation already showed signs of stress in 2001. The same can be seen in Figure 9, showing high vegetative vigor in 2000, followed by a slight decrease in 2001 and a significant decrease in 2007, with about 30,000 m^2^ in 2022.

Nevertheless, as the NDVI was not created explicitly for detecting hydrothermal anomalies, the future development of this methodological approach might benefit from using simultaneous ratio bands, other sensors’ data (namely, nighttime LANDSAT data, which currently are not available for the Azores archipelago) with higher spatial resolutions, and other visible wavelengths to create thresholds able to identify volcanic-related changes. Using more wavelengths in addition to thermal infrared might constitute an effective methodological approach for monitoring hydrothermal systems since the case study’s results show that the spatial resolutions of the thermal bands are not high enough to detect changes in a shorter period. In this particular case, the vegetation in the study site was a positive aspect, since the vegetation vigor changes contributed to identifying the thermal anomalies. A similar non-vegetated area could be more challenging to identify due to the dimensions of the thermal anomaly.

## 5. Conclusions

As far as we know, this is the first time thermal RS images have been tested in the Azorean fumarolic fields. The selected test site resulted from a new thermal anomaly that extended the Caldeiras da Ribeira Grande geothermal area, in the north flank of Fogo Volcano (São Miguel Island), after 2010.

Applying RS techniques to hydrothermal areas (maximum 100 °C) is challenging, as shown in other similar degassing areas (e.g., the Nisyros, Solfatara, and Yellowstone volcanoes). The main objective of this research was to analyze the effectiveness of the ASTER data for identifying surface changes in hydrothermal systems after the appearance and development of a thermal anomaly at the Caldeiras da Ribeira Grande geothermal area. The ASTER data showed promising results, as it was possible to identify the thermal anomalies with band 13 (90 m of spatial resolution) around one year and a half after the in situ visible manifestations. Nevertheless, using near-infrared and red bands (15 m spatial resolution) for the NDVI map computation, it was possible to detect the surface changes within four months, highlighting the importance of using both thermal and visible multispectral images to detect thermal anomalies in hydrothermal systems.

This case study confirms the need for new thermal sensors with higher spatial and temporal resolutions to increase the effectiveness of early detection of anomalies in volcanic areas. Although the current study highlighted the high strategic relevance of in situ measurements, this methodological approach effectively identified the thermal anomaly. Application of this methodology to other hydrothermal areas may constitute a complementary tool for the volcano monitoring observatories.

## Figures and Tables

**Table 1:** The ASTER bands features [29].

Operational Dates	Band	Reflected Range (µm)	Spatial Resolution (m)	Band Explanation/Uses
Dec 1999 to Present	1	0.52–0.60	15 m	Visible and Near-Infrared
	2	0.63–0.69	15 m	Visible and Near-Infrared
	3N	0.78–0.86	15 m	Visible and Near-Infrared
Dec 1999 to April 2008	4	1.600–1.700	30 m	Shortwave Infrared
	5	2.145–2.185	30 m	Shortwave Infrared
	6	2.185–2.225	30 m	Shortwave Infrared
	7	2.235–2.285	30 m	Shortwave Infrared
	8	2.295–2.365	30 m	Shortwave Infrared
	9	2.360–2.430	30 m	Shortwave Infrared
Dec 1999 to Present	10	8.125–8.475	90 m	Thermal Infrared
	11	8.475–8.825	90 m	Thermal Infrared
	12	8.925–9.275	90 m	Thermal Infrared
	13	10.25–10.95	90 m	Thermal Infrared
	14	10.95–11.65	90 m	Thermal Infrared

**Table 2:** Satellite images considered for the analysis.

Data Acquisition Dates	(Day/Night)
25 February 2010	Day
29 March 2010	Night
23 April 2010	Day
24 June 2010	Night
13 August 2010	Day
5 September 2010	Day
21 September 2010	Night
16 March 2011	Day
14 August 2011	Night
12 April 2012	Day
9 August 2012	Night

**Table 3:** Classification of NDVI ranges.

NDVI Ranges	Class of Vegetation Vigor
−1–0.2	Very Low
0.2–0.4	Low
0.4–0.6	Moderately Low
0.6–0.8	Moderately High
0.8–1	High

## Data Availability

The remote sensing data used in this study are openly provided by the US Geological Survey (USGS), Earth Resources Observation and Science (EROS) Data Center archive (https://earthexplorer.usgs.gov, accessed on 22 February 2022), or upon request from the corresponding author. The in situ fieldwork data used in this research are the property of IVAR/CIVISA.
